# Production by Tobacco Transplastomic Plants of Recombinant Fungal and Bacterial Cell-Wall Degrading Enzymes to Be Used for Cellulosic Biomass Saccharification

**DOI:** 10.1155/2015/289759

**Published:** 2015-06-02

**Authors:** Paolo Longoni, Sadhu Leelavathi, Enrico Doria, Vanga Siva Reddy, Rino Cella

**Affiliations:** ^1^Dipartimento di Biologia e Biotecnologie, Università di Pavia, Via Ferrata 9, 27100 Pavia, Italy; ^2^Dipartimento de Biologie Végétale, Université de Geneva, 30 Quai Ernest Ansermet, Sciences III, 1211 Genève, Switzerland; ^3^Plant Transformation Group, International Center for Genetic Engineering and Biotechnology, Aruna Asaf Ali Marg, New Delhi 110067, India; ^4^Centre of Sustainable Livelihood (CSL), Vaal University of Technology, Vanderbijlpark 1900, South Africa

## Abstract

Biofuels from renewable plant biomass are gaining momentum due to climate change related to atmospheric CO_2_ increase. However, the production cost of enzymes required for cellulosic biomass saccharification is a major limiting step in this process. Low-cost production of large amounts of recombinant enzymes by transgenic plants was proposed as an alternative to the conventional microbial based fermentation. A number of studies have shown that chloroplast-based gene expression offers several advantages over nuclear transformation due to efficient transcription and translation systems and high copy number of the transgene. In this study, we expressed in tobacco chloroplasts microbial genes encoding five cellulases and a polygalacturonase. Leaf extracts containing the recombinant enzymes showed the ability to degrade various cell-wall components under different conditions, singly and in combinations. In addition, our group also tested a previously described thermostable xylanase in combination with a cellulase and a polygalacturonase to study the cumulative effect on the depolymerization of a complex plant substrate. Our results demonstrate the feasibility of using transplastomic tobacco leaf extracts to convert cell-wall polysaccharides into reducing sugars, fulfilling a major prerequisite of large scale availability of a variety of cell-wall degrading enzymes for biofuel industry.

## 1. Introduction

Biofuels are currently obtained from edible vegetable products (sucrose, starch, and triglycerides), but ethical considerations as well as problems of economic sustainability have stimulated the development of second and third generation biofuels derived from nonedible cellulosic biomass and lipogenic unicellular algae [1, 2]. The conversion of plant biomass and cultivation waste (Agri-Waste) into bioethanol is considered a sustainable process as it (1) reduces the dependency on fossil fuels like coal- and petroleum-based products, (2) reduces the negative impact on the environment being a carbon-neutral cycle, (3) allows us to obtain secondary byproducts with application in pharmaceutical and biotechnological industries from the residual biomass. The plant cell wall is a complex structure consisting of a mixture of cellulose, hemicelluloses, and lignin, varying from plant to plant. Cellulose is the most diffuse source of reduced carbon in the world, ranking second only to fossil carbon [3]. In order to convert plant biomass into biofuels, cell-wall macromolecules must be depolymerized to sugar monomers that can be fermented to ethanol or other alcohols with a higher number of carbons through the action of yeast or bacterial strains. Alternatively they can be used as growth substrate for lipogenic microorganisms to obtain lipid to be later transformed in biofuel by different treatments. The current technology adopted to degrade cellulose uses high energy-consuming approaches in order to destroy its stable paracrystalline portion. Several fungi and bacteria synthesize all the enzymes required to degrade cell-wall polysaccharides to simple sugars or oligosaccharides from which they obtain the energy to support their growth. Some of these microorganisms are thermotolerant and possess enzymes active at medium-high temperatures (60°C), reviewed in [4, 5]. A particularly important need is the availability of large amounts of suitable enzyme cocktails for the saccharification of huge amounts of cellulosic residues and wastes. Current estimates suggest that about 225 M tons of cellulosic biomass/year are available in EU alone [6]. The cost of enzymes used for saccharification is one of the three crucial parameters for the economical sustainability of biofuel production [7, 8].

A number of bacterial and fungal strains able to depolymerize plant cell walls have been described and characterized [9, 10]. However, the expression level of these enzymes by wild-type strains is generally low. The recombinant DNA technology in combination with improved bioreactors has been shown to increase significantly the production of microbial enzymes. Prokaryotic and eukaryotic expression systems based on recombinant DNA approaches have been employed for the production of proteins/enzymes of commercial interest and their advantages and disadvantages evaluated [5, 11–14]. Enzymes used for industrial applications, among which biofuel production is found, are currently produced* via* microbial fermentation even if the process requires high investment, production, and maintenance costs. Several studies show that protein/enzyme production by plant molecular farming might offer some advantages over microorganisms, as plants have both eukaryotic (nuclear) and prokaryotic (chloroplast) expression systems [15–18] that can be used singly or in combination. Transgenic plants were shown to be a valuable system for the production of a variety of antibodies, proteins/enzymes, and vaccines [19]. A large number of genetically modified crops expressing genes encoding insecticidal proteins and enzymes conferring resistance to herbicides are grown all over the world [20]. However, the production of recombinant proteins/enzymes based on nuclear transformation remained a major limitation as the level of recombinant proteins accumulation is generally low. Conversely, a chloroplast-based expression system offers several advantages with respect to the molecular farming notion. Plastid genome (plastome), being prokaryotic in origin, uses operons for the expression of multiple foreign genes under a single promoter. As the integration of transgene constructs takes place through homologous recombination, there is a unique transformation event without any positional effects, contrary to what is observed in the case of nuclear transformation due to random integration of foreign genes into the nuclear genome. Due to independent plastidial transcription, translation, and protein folding machineries, recombinant genes were generally shown to be expressed in chloroplasts at levels higher than that achieved with nuclear-based expression systems [21]. In most plant species, among which* Nicotiana tabacum*, the plastome is inherited maternally thus avoiding transgene dispersion by pollen. Moreover, tobacco, being a nonfood and nonfeed plant, is ideal as a recombinant protein expression system since it does not mix with the food chain, a major issue for regulatory clearances for commercial activities [22]. The low cultivation cost and ease of up-scale production of transplastomic plants (plants with transformed plastid genome) by simply increasing the cultivation area provide additional advantages. More than a decade ago, Leelavathi et al. [15] were the first to demonstrate the feasibility of accumulating a bacterial thermostable xylanase, which has several industrial applications including the biofuel industry, using a chloroplast genetic engineering approach. Later, this approach has been used to express a large number of cellulolytic enzymes [16, 23–26]. Besides pointing to chloroplast transformation as a promising technology for the large scale production of recombinant enzymes, the study of Leelavathi et al. [15] also showed that the plant produced recombinant xylanase retained all biochemical functions, similarly to the native bacterial one. It is also noteworthy that thermostability of recombinant enzymes is a crucial feature since it allows us to partially overlap the cellulose pretreatment process with its digestion.

In the present work we expressed in tobacco chloroplasts five cellulase genes isolated from different microbial organisms and a polygalacturonase gene from* Aspergillus niger*. Leaf extracts containing the recombinant enzymes were tested for their ability to degrade various cell-wall components under different conditions, singly and in combinations. Also the previously described thermostable xylanase [15] was used in combination with cellulases and a polygalacturonase to study the cumulative effect on the depolymerization of complex plant biomass. Our results demonstrate the feasibility of converting cell-wall polysaccharides into reducing sugars using a combination of tobacco cell extracts containing enzymes with compatible temperature and pH optima.

## 2. Materials and Methods

### 2.1. Chemicals

All the reagents and chemicals were purchased from Sigma-Aldrich (St. Louis, MO, USA).

### 2.2. Construction of Chloroplast Plastid Transformation Vectors for the Over Production of Cellulolytic Enzymes in Tobacco

**Figure 1:**
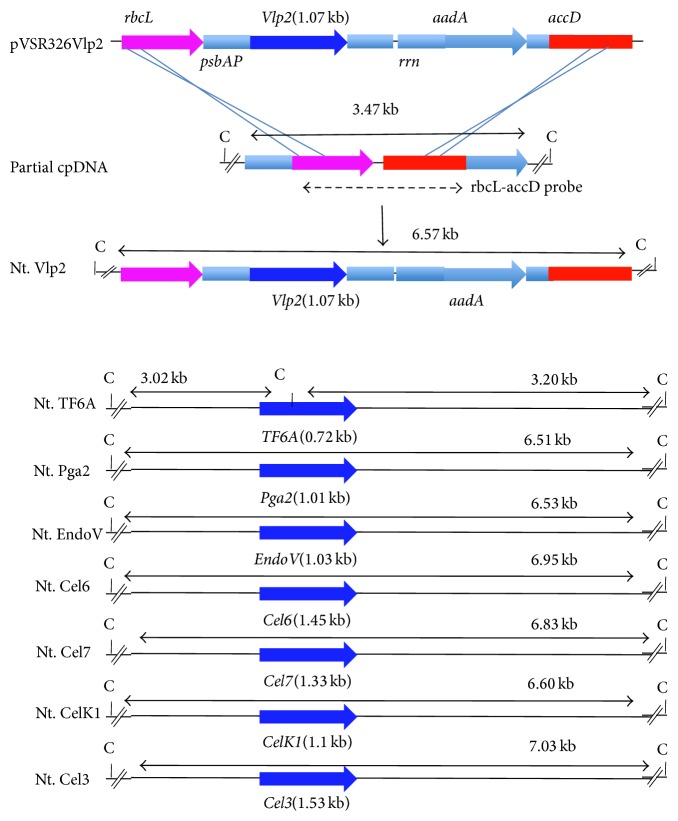
Portion of the chloroplast transformation vector map containing the gene coding for Vlp2 (pVSR326Vlp2), integration site of tobacco chloroplast DNA (cpDNA), and the same region in transplastomic tobacco plants are shown. Also restriction map of vectors containing genes coding for other cellulolytic enzymes is shown. All the genes coding for cellulolytic enzymes were put under the expression signals of rice* psbA*. Lines with double arrow indicate the size of DNA fragments after the restriction digestion with* Cla*I restriction enzyme. Dashed arrow indicates the FLK probe (*rbcL*-*aacD* flanking region) used to confirm site specific integration of transgenes. A possible mechanism for site-specific integration of* aadA* and* Vlp2* through two homologous recombination events (crossed lines) is also shown. Size of the coding region of each gene is shown in brackets.

The plastid transformation vector pVSR326 (Figure 1, GenBank acc. number AF527485) was used to clone all genes used in the study. pVSR326 vector contains the* aadA* coding sequence, which confers resistance to both spectinomycin and streptomycin, under the constitutive 16S rRNA promoter and with the terminator of* rbcL* [15, 21].

The DNA sequences of genes encoding the enzymes used in the present study stored in the GenBank are GH6 CHGG_10762 (*Cel6*, exoglucanse) and* gh7* CHGG_08475 (*Cel7*, endoglucanase), GH45 (*EndoV*, endoglucanase) CHGG_08509 of* Chaetomium globosum* [27], GH 5 (*CelK1*, endoglucanase) (GenBank acc. number AAL83749) from* Paenibacillus* sp. KCTC8848P; GH7_CBH-EG* Cel3*, exo-cellobiohydrolase from* Phanerochaete chrysosporium* (AAB46373);* TF6A* (GenBank acc. number M73321);* Pga2* (GenBank acc. number XM_001397030);* Vlp2* peroxidase (GenBank acc. number XM_001220787). For cloning into transformation vector, gene sequences were either amplified by polymerase chain reaction (PCR) using the primers indicated in Table 1 or got synthesized based on protein sequence.

Alternatively, on the basis of the amino acid sequence available for* celK1* (GenBank acc. number AAL83749) we designed synthetic cDNA according to tobacco chloroplast “codon usage” (http://www.kazusa.or.jp/codon/) to optimize synthesis and accumulation of the relevant enzyme. All sequences were cloned at* Nco*I and* Sac*I sites of pVSR326 (Genbank acc. No. AF527485) by replacing the* uidA* (GUS) reporter gene. Sequences containing an internal* Nco*I restriction site (i.e.,* Pga2*) were cloned in two steps. In the first step the C-terminal end of the* Pga2* was cloned as* Nco*I-*Sac*I fragment and then the N-terminal end of the genes was cloned as* Nco*I-*Nco*I fragment. The orientation of the ATG in relation to the C-terminal part was confirmed by PCR and sequencing. All the genes are placed under the* psbA* gene regulatory elements.

### 2.3. Plant Transformation and Molecular Analysis

Transplastomic tobacco (*Nicotiana tabacum* cv. Petit Havana) plants were obtained using the particle delivery method described earlier [15]. Bombarded leaf explants (T0) were regenerated on selective RMOP medium containing spectinomycin (500 mg/L). Regenerated green shoots obtained 30 days after bombardment were grown to maturity to collect seeds (T1) that were germinated on agar plates containing spectinomycin and streptomycin (500 mg/L, each). In order to obtain homotransplastomic lines, T1 leaf explants were cultured on RMOP medium containing spectinomycin and streptomycin (500 mg/L, each). This process was repeated up to three times (T3).

Southern blot analysis was used to confirm site-specific integration of transgenes and homoplasticity of transplastomic plants. Total genomic DNA was isolated using the Trizol method (Sigma-Aldrich, USA), digested with* Cla*I, separated on 0.8% agarose gel and blotted onto Nylon membranes that after UV irradiation were probed with ^32^P labeled DNA corresponding to* rbcL-accD* DNA flanking region and to the coding region of the genes of interest. Northern blot analysis was carried out to confirm efficient transcription of all tested genes. In both cases standard procedures were followed for hybridization and washing [28].

### 2.4. Protein Extraction and Enzyme Activity

Following a preliminary screening of activity with leaves of different age, fully expanded leaves were used to extract the enzymes of interest. Crude leaf homogenates were used in all cases, in view of developing a simple and cost-effective industrial saccharification process. A 1 g leaf sample from each transplastomic plant was cut into small pieces and ground in a mortar with liquid nitrogen and 3 mL of extraction buffer added to the resulting powder. Acetate or phosphate buffer was used in the 4.0–8.0 pH range as indicated. The plant homogenate was then mixed and centrifuged for 10 min at 16,873 ×g and collected the supernatant in a new Eppendorf tube. In order to eliminate the presence of the endogenous sugars that may subsequently interfere with the reducing sugar assay and to concentrate it, the leaf extract was filtered using Vivaspin 500 (28-9322-18) columns with a cut-off of 3 kDa. The concentration of total soluble protein (tsp) was determined using the Bradford reagent (Sigma-Aldrich, USA) according to the manufacturer's instructions. For all enzyme assays a concentration of 0.1 mg/mL of total soluble protein (tsp) content was used.

### 2.5. Preparation of the Poplar Wood Powder

The poplar wood samples used for laboratory analysis are represented from branches and stem of a poplar clone supplied by the “Franco Alasia Vivai” company, Savigliano (Cuneo), Italy. Before conducting the experiments, the wood samples were dried overnight at 40°C and then cut into small pieces (length 0.5–1 cm; width 2-3 mm; height 1-2 mm) using vineyard scissors. Wood chips were then ground to fine powder using the mill MM301 from Retch at the frequency of 30 vibrations/sec for 20 seconds, repeating each cycle for three times.

### 2.6. Enzymatic Activity Assays

Cellulase activity was assayed, incubating for 60 min at different temperatures, in 1 mL of the plant extract (0.1 mg/mL, tsp) containing 0.02 g of carboxymethylcellulose (CMC) or microcrystalline cellulose (MCC); xylanase activity was determined incubating the same extract with 0.02 g of xylan in the same experimental conditions used for the previous assay; the same procedure was adopted to test the polygalacturonase activity using polygalacturonic acid or apple pectin as substrates. In order to assess the total hydrolytic activity of the leaf extract, 0.02 g of the wood powder was incubated with 1 mL of plant extract; the amount of released reducing sugars was determined by dinitrosalicylic acid (DNS) method [29]. A fraction of the incubated extract (250 *μ*L) was added to 250 *μ*L of water and to 1.5 mL of DNS reagent in a 2 mL test-tube, boiled for 10 minutes, and then cooled down at room temperature. Sample absorbance at 540 nm was recorded against a water-DNS mixture blank. A glucose calibration curve (0.2–0.5 mg/mL) was used to determine the amount of reducing sugars (mg/g of substrate) after the reaction. Celk1 cellulase activity was tested also using a filter paper as a substrate: disks of filter paper (5 mm of diameter) were incubated at the indicated temperature with 3 mL of the plant extract (pH 5) containing 0.1 mg/mL of protein content. The amount of sugars released was determined by DNS assay after 1.5, 6, and 20 hours of incubation. A control sample was prepared incubating the paper sheet with the same volume of acetate buffer (pH 5). Peroxidase activity of VPL2 was determined spectrophotometrically at 610 nm monitoring the oxidation of phenol red [30].

## 3. Results

### 3.1. Vector Construction for the Transformation of Tobacco

Sequences encoding cell-wall degrading enzymes derived from different sources were cloned into pVSR326 vector by replacing the coding region of reporter* uidA* gene with the sequence of interest (Figure 1). pVSR326 vector integrates the transgene cassette into the Single Large Copy region between* rbcL* and* accD* noncoding region in a site specific manner [21]. The recombinant gene encoding the enzyme of interest was placed under the regulation of chloroplast-specific* psbA* gene promoter and terminator (Figure 1). The native* rbc*L*-acc*D region was used as flanking regions for a site-specific integration of transgenes through two possible homologous recombination events (Figure 1). The pVSR326 contained a selectable* aadA* gene conferring resistance to spectinomycin/streptomycin. The direction and the size of the expected transcripts for all the genes are shown in Figure 1.

### 3.2. Production of Stable Tobacco Transplastomic Plants

**Figure 2:**
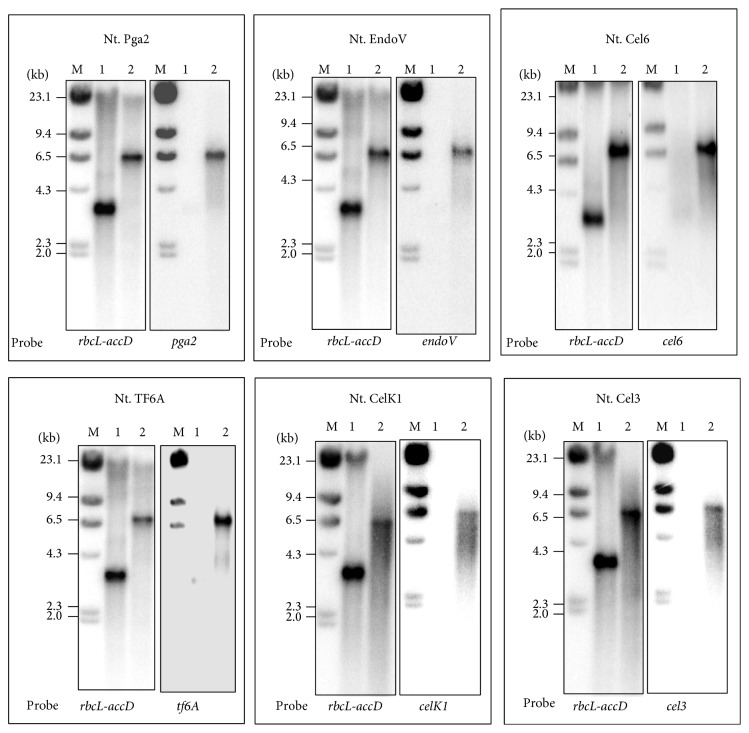
Southern blot hybridization to show site-specific integration of introduced transgenes into tobacco plastid genome for the representative transplastomic plant. The partial coding region of* rbcL* and* accD* was used to show the stable and site specific integration of transgenes. Gene specific DNA probe was also used to confirm the stable integration of the transgene. Note the lack of any untransformed plastid DNA in the transplastomic lines. Molecular marker (M), wild type (1), and transformed (2) plants.

**Figure 3:**
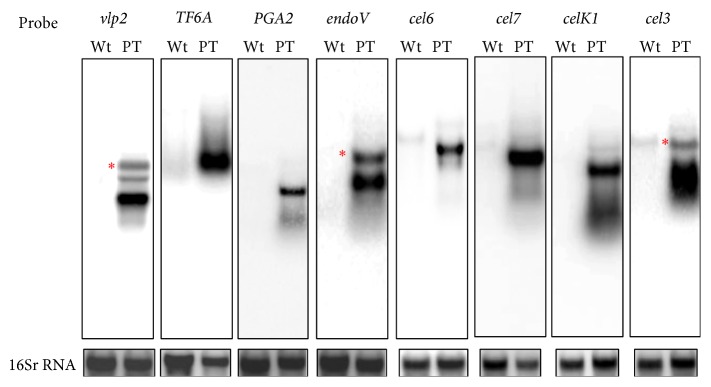
Northern blot analysis showing the expression of transgenes in tobacco chloroplasts. RNA isolated from untransformed control (Wt) and plastid transformed (PT) was separated on formaldehyde-agarose gels, blotted on to the Hybond-N+ membrane, and hybridized with gene specific probes. For the loading control, the same blots were hybridized again with 16S rRNA probe (lower panel): (1) Nt. Vlp2, (2) Nt. TF6A, (3) Nt. Pga2, (4) Nt. EndoV, (5) Nt. Cel6, (6) Nt. Cel7, (7) Nt. CelK1, and (8) Nt. Cel3. The red asterisks indicate the putative longer transcript initiated by the upstream* rbcL* promoter element.

The Bio-Rad Biolistic PDS-1000/He Particle Delivery System was used to transform tobacco chloroplasts [21]. Transplastomic plants were selected under spectinomycin containin medium [15]. Out of 20 leaves bombarded with each construct, about 30–45 green shoots were obtained, 30 days after bombardment on RMOP selection medium. In order to obtain homotransplastomic lines, leaf explants from the regenerated plants were subcultured again on selective RMOP medium. This process was repeated up to three times and the degree of homoplasticity was assessed by southern hybridization. One of cellulase-producing lines T3 lines,* cel3*, turned white and lost its ability to grow autotrophically. Interestingly, these plants could be maintained in the greenhouse in a heteroplasmic state (see Figure S1 in Supplementary Material available online at http://dx.doi.org/10.1155/2015/289759). Severe pleiotropic effects were also observed with plants expressing* Bgl1C*,* Cel6B*,* Cel9A*, and* Xeg74* genes from* Thermobifida fusca* [25] and therefore these lines were not further considered. Southern blot hybridization was used to prove stable and site specific integration of transgenes and the selectable* aadA* gene into the tobacco plastid genome. Hybridization with the flanking region (*rbcL-accD* probe) has confirmed site-specific integration of transgenes into the intergenic region between* rbcL* and* acc*D genes (Figure 2). Absence of any band corresponding to the low molecular weight band observed in the wild type plants is a clear indication for the homotransplastomic nature of their plastome. The stable integration of transgenes into plastid genome was further confirmed by reprobing the blots with gene specific coding sequences as probes. An expected size band was observed in all the transformed plants (Figure 2). The* aadA* gene that confers resistance against spectinomycin and streptomycin was used again to test the progeny for stable inheritance of the transgenes in the T1 generation. All seedlings derived from seeds produced after self-pollinatination are expected to remain green when germinated on plates containing both spectinomycin and streptomycin, if the progeny inherit the selectable* aadA* gene [21]. When the seeds obtained after self-pollination of T0 generation plants were germinated on the agar plates containing both spectinomycin and streptomycin, all seedlings remained green while the seedlings from the wild type untransformed plants turned white, providing evidence for the stable integration and inheritance of the transgenes by the progeny plants (data not shown). Furthermore, northern blot analysis confirmed efficient transcription of transgenes since transcripts of the expected size were found in all the transplastomic plants analyzed (Figure 3). The intensity of the transcript bands suggests efficient transcription of transgenes under* psbA* gene regulatory elements in tobacco chloroplasts. In some cases, in addition to the expected size of transcripts, additional minor bands of higher molecular weight were observed. These might represent transcripts of the same transgenes arising from the* rbcL gene* promoter present upstream to the site of transgene integration.

### 3.3. Expression of Cell-Wall Degrading Enzymes in Chloroplasts and Their Biochemical Properties

In order to assess the activity of the chloroplast-accumulated enzymes, crude extracts obtained from healthy tobacco plants were tested using commercially available substrates or raw wood.

**Figure 4:**
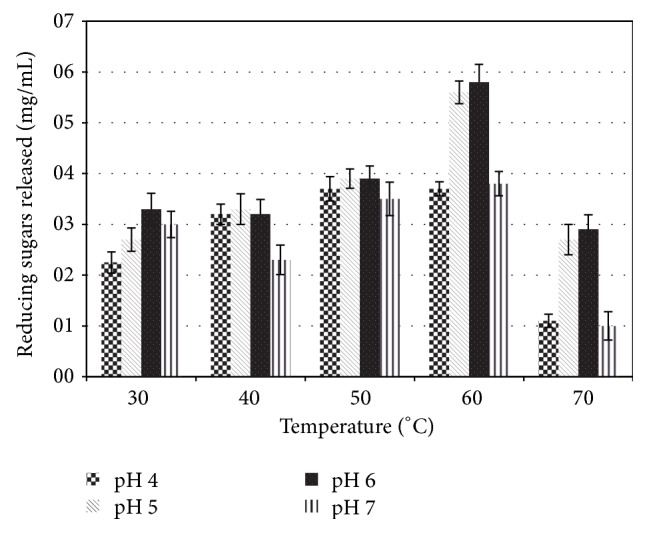
Reducing sugar released by the recombinant endoglucanase Nt CelK1 in different temperature and pH conditions using CMC as a substrate. The final protein concentration in each assay was 0.1 mg/mL. The activity values are expressed as mg/mL of reducing sugars assayed by the DNS method.

Among T3 generation plants, those producing CelK1 showed the highest cellulase activity at 60°C in a pH range of 5.0–6.0 and using CMC cellulose as a substrate (Table 2). However, as shown in Figure 4, the amount of reducing sugars released dropped considerably when the temperature was raised to 70°C. Optimal CelK1 enzyme activity was observed at pH 6.0 and 60°C (Figure 4). As for the NT. Vlp2 transplastomic plants, we failed to detect peroxidase activity in leaf homogenates and therefore this transformant was not further considered.

**Figure 5:**
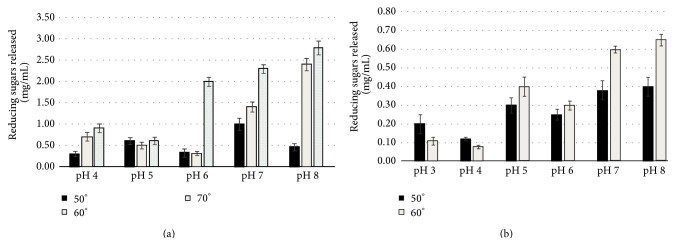
Activity of the recombinant polygalacturonase Nt Pga2 in different pH and temperature conditions using different substrates: (a) apple pectin (Sigma-Aldrich), (b) raw poplar wood. The final protein concentration in each assay was 0.1 mg/mL. The activity values are expressed as mg/mL of reducing sugars.

The transplastomic Nt. Pga2 plant expressing* Pga2* showed significant pectinase activity when its leaf extract was tested on apple pectin substrate. The most efficient Pga2 activity was observed in the 6.0–8.0 pH range and at a temperature ranging between 60°C and 70°C; in particular the polygalacturonidase activity was higher at highest temperature and basic conditions (Figure 5(a)). The amount of reducing molecules (galacturonic acid monomers or oligogalacturonides) released at 70°C and pH 8.0 was more than four times the amount of those released at 50°C and pH 7.0, suggesting that the Pga2 is a thermostable enzyme that retained its activity when produced in tobacco chloroplasts (Figure 5(a)). Even when Pga2 was tested using raw popular wood as a substrate, a very high activity was observed at 60°C and pH 8.0 (Figure 5(b)). On the other hand, despite the efficient transcription, no detectable cellulase activity was observed in the plants transformed with* Cel6*,* Cel7*,* TF6A*, and* EndoV* genes.

### 3.4. A Combination of CelK1 and Xylanase (BSX) or Pga2 with Similar Thermostable Properties Improves the Depolymerization of a Complex Cellulosic Biomass

**Figure 6:**
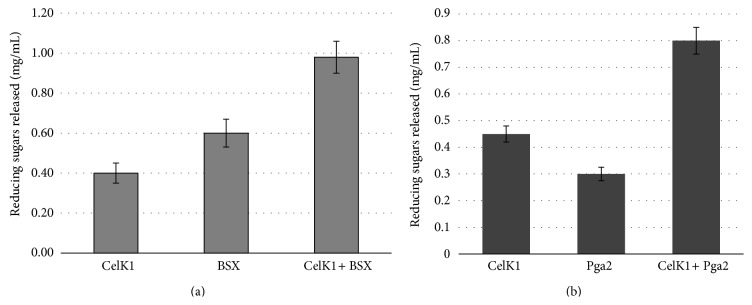
Activity of recombinant Nt CelK1 in combination with recombinant Nt BSX (a) or recombinant Nt Pga2 (b) on raw poplar wood as substrate. The final protein concentration in each assay was 0.1 mg/mL. The activity is expressed as mg/mL of reducing sugars released.

**Figure 7:**
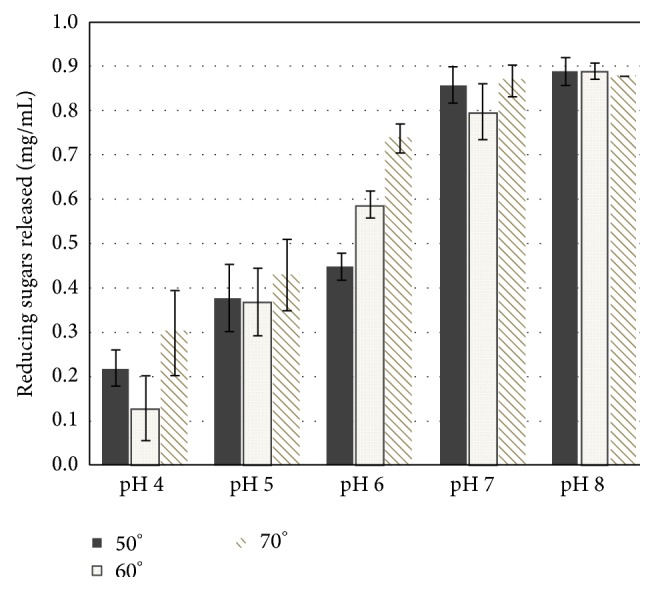
Activity assays of the enzymatic cocktail composed by recombinant Nt BSX, Nt CelK1, and Nt Pga2 in different pH and temperature conditions. Raw popular wood was used as substrate. The activity is expressed as the concentration of reducing sugar released.

To study the depolymerization of a complex substrate such as poplar wood powder we tested a combination of enzymes in different temperature and pH conditions. CelK1 leaf extract was used in combination with a homogenate obtained from either a previously described line overexpressing a thermostable xylanase (BSX) [15, 18] or a* Pga2* transformed line. Since the final protein concentration in each assay was 0.1 mg/mL, the amount of reducing sugars released from poplar wood when assayed at pH 7 with a mixture of CelK1 and BSX was synergistic as compared to the action of each enzyme alone (Figure 6(a)). The same synergistic effect was also observed when raw wood powder was exposed to the action of a combination of CelK1 and Pga2 (Figure 6(b)). As compared to CelK1 alone, the amount of reducing sugars released increased by more than twofold when Pga2 was present in the reaction mixture. These results cannot be explained only by the fact that the two enzymes use different substrates but rather suggest that the removal of pectin or xylan makes cellulose more accessible to Celk1. On the basis of these encouraging results we tested a mixture of the three enzymes (BSX, Pga2, and CelK1) for the ability to release reducing sugars. As shown in Figure 7, the best results were obtained at 70°C and pH 8. However, since the temperature optimum of CelK1 is 60°C, it might be advisable to perform the digestion of the biomass in two steps: treat the raw wood powder first with BSX and/or Pga2 at 70°C, and then add Celk1 and continue the incubation at 60°C. As far as the temperature is concerned, this is a particular interesting result since at the industrial process for the production of bioethanol, the woody biomass is subjected to a heat treatment of over 100°C (steam explosion) before the enzyme addition and thus the possibility of adding cell-wall degrading enzymes at 70°C might effectively contribute to reduce the saccharification time and contribute to speed up the industrial process.

## 4. Discussion

Expression of cell-wall degrading enzymes in plants using a nuclear-based transformation approach is a major challenge as the cellulolytic enzyme(s) can interact with the plant cell wall and thereby interfere with cell growth and plant development [31]. To prevent potentially harmful consequences caused by recombinant cell-wall degrading enzymes, a number of strategies were evaluated among which targeting to subcellular compartments [32], rhizosecretion into hydroponic culture medium [33], and accumulation of a fusion storage proteins in seed oil bodies [34]. However, all these approaches are characterized by a low expression of recombinant enzymes generally associated with nuclear transformation and expression system. Thus, chloroplast transformation was deemed more suitable to obtain a high level of accumulation of recombinant proteins. Although chloroplast transformation offers the possibility of polycistronic transcription, we chose to express a single enzyme per transplastomic plant for two main reasons. First, single cell-wall degrading enzymes find large industrial application. For instance, cellulases are used in the textile industry (stone-washing), [35], while xylanases are used for pulp whitening and animal feed processing [36]. Moreover, the availability of a repertoire of single enzymes allows a better formulation of the most suitable cocktail optimal for each lignocellulosic biomass available (woody biomass, grasses, wastepaper, etc.).

Secondly, whenever an enzyme cocktail is required, the availability of single enzymes offers the possibility to plan the timely addition of different enzymes. For instance, the efficiency at which cell-wall cellulose can be digested will be improved if the biomass is pretreated with a polygalacturonidase before the addition of cellulases. In fact, a combination of CelK1 and PGA2 enzymes showed an additive effect on the release of reducing sugars from poplar wood (Figure 6(b)). Interestingly, when both CelK1 and PGA2 were used together the amount of reducing sugars released increased by more than twofold those suggesting that the removal of pectin by PGA2 is making cellulose more accessible to CelK1.

A third important reason to avoid a simultaneous multiple expression of several genes refers to a possible incompatibility of accumulation of a given protein with chloroplast physiology. In fact, it was observed that plants singly expressing* bgl1C*,* cel6B*,* cel9A*, and* xeg74* genes from* T. fusca* showed severe pleiotropic effects [25]. Therefore, the interference of a single protein with chloroplast biogenesis and/or stability of the photosynthetic apparatus might hamper the expression of the remaining ones.

In the biorefinery process for the production of bioethanol, a pretreatment of plant biomass is required to make cell-wall polymers more accessible to the enzymes required for their deconstruction [37]. Although energy-consuming, such pretreatment, is necessary to reduce the amount of enzymes, which represent the most relevant cost of the entire process [38]. It is tempting to speculate that plant molecular farming, due to the ease of large scale production of recombinant enzymes, might effectively contribute to reduce the saccharification cost.

In conclusion, this study proves that a combination of three enzymes targeting different components of the plant cell wall but having compatible temperature and pH optima not only improves the saccharification of cellulose present in a complex plant biomass but also reduces the number of steps involved in the downstream processing. Our future endeavor would include identification of factors involved in the low or lack of expression/accumulation of beta-glycosidase (*Bgl*) and also identify* Bgl* genes from other sources having suitable biochemical properties, in order to improve further the cellulosic biomass saccharification.

## Supplementary Material

Figure S1: Homoplastidial and heteroplastidial transplastomic tobacco plants expressing cel3 gene. The plasmid DNA present in the white sectors of both types of plants was extracted and analyzed by Southern blot hybridization using various probes. This analyses indicated the occurrence of deletion/rearrangements in the plastome of white cells.

## Figures and Tables

**Table 1:** 

Gene	Forward primer (sequence from 5′-3′)	Reverse primer (sequence from 5′-3′)
*Cel7 *	TGCTACATCACCCCCTTCAT	GTACTTGCGGTGGATGGACT
*Cel6 *	GATGTGGGCCAACGACTACT	GTGGATGGTCAGCTCCTTGT
*Cel3 *	ATGGCACAGCAGGCAGGTACAC	ATAACACTGGCTGTAATACGGATTC
*CelK1 *	ATGGCCAGCGTTAAAGGTTATTACC	TTCTGCTGCTGCTTTTGCCTGTTCTGC
*EndoV *	TACGCCATGGCTCGCTCTACTCCCATTCTTCG	AGCTGAGCTCTTAAAGGCATTGCGAGTACCAGTCG
*Pga2 *	ATGGACAGCTGCACGTTCACC	CTAACAAGAGGCCACCGAAGG
*Vlp2 *	ATGTCGACCGCAACTCGCACTTTC	TTAGGCGTTGACGGTCTTGAACAC
*TF6A *	ATGTCCCCCAGACCTCTTCGC	TCAGCTGGCGGCGCAGGTAAG

**Table 2:** Activity of various tobacco chloroplast expressed enzymes on carboxymethylcellulose (CMC) and on microcrystalline cellulose (MCC) substrates.

Enzyme	Activity U/mg protein		Type	Generation of plants used in the study
	CMC	MCC		
Cel63	0.3 ± 0.02	0.5 ± 0.03	Exoglucanase	T3
Cel3	0.36 ± 0.02	0.12 ± 0.005	Endoglucanase	T3
CelK1	3.6 ± 0.15	0.2 ± 0.03	Endoglucanase	T3
Cel6	0.16 ± 0.03	0.4 ± 0.05	Exoglucanase	T1
Cel7	0.25 ± 0.009	0.21 ± 0.03	Endoglucanase	T1
