# Supplementary material for: Production by Tobacco Transplastomic Plants of Recombinant Fungal and Bacterial Cell-Wall Degrading Enzymes to Be Used for Cellulosic Biomass Saccharification
Source: Biomed Res Int. 2015 Jun 2;2015:289759. doi: 10.1155/2015/289759 (PMC4468278; doi:10.1155/2015/289759)
Supplement: Supplementary file 1 — Figure S1: Homoplastidial and heteroplastidial transplastomic tobacco plants expressing cel3 gene. The plasmid DNA present in the white sectors of both types of plants was extracted and analyzed by Southern blot hybridization using various probes. This analyses indicated the occurrence of deletion/rearrangements in the plastome of white cells. [file 289759.f1.pdf]

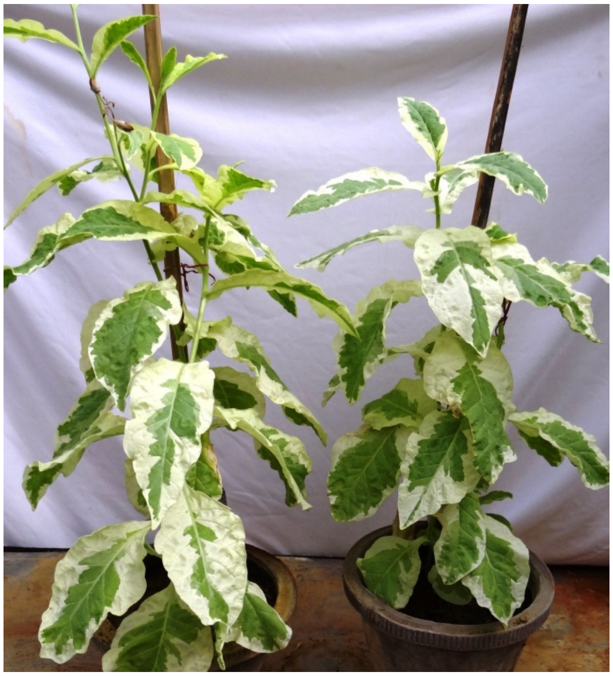

Longoni et al figure S1

Supplementary figure (S1) legends

Figure S1. Homoplastidial and heteroplastidial transplastomic tobacco plants expressing *cel3* gene. Possible deletion/rearrangements in the plastid DNA in the white sectors was verified using various probes in Southern hybridization.
